# Folate receptor‐targeted aminoglycoside‐derived polymers for transgene expression in cancer cells

**DOI:** 10.1002/btm2.10038

**Published:** 2016-10-21

**Authors:** Sudhakar Godeshala, Rajeshwar Nitiyanandan, Brian Thompson, Sheba Goklany, David R. Nielsen, Kaushal Rege

**Affiliations:** ^1^ Chemical Engineering Arizona State University Tempe AZ 85287; ^2^ Biological Design Program Arizona State University Tempe AZ 85287

**Keywords:** competitive inhibition, folate receptor, folic acid, gene delivery, neomycin, paromomycin, transgene expression

## Abstract

Targeted delivery of anticancer therapeutics can potentially overcome the limitations associated with current chemotherapeutic regimens. Folate receptors are overexpressed in several cancers, including ovarian, triple‐negative breast and bladder cancers, making them attractive for targeted delivery of nucleic acid therapeutics to these tumors. This work describes the synthesis, characterization and evaluation of folic acid‐conjugated, aminoglycoside‐derived polymers for targeted delivery of transgenes to breast and bladder cancer cell lines. Transgene expression was significantly higher with FA‐conjugated aminoglycoside‐derived polymers than with Lipofectamine, and these polymers demonstrated minimal cytotoxicty. Competitive inhibition using free folic acid significantly reduced transgene expression efficacy of folate‐targeted polymers, suggesting a role for folate receptor‐mediated uptake. High efficacy FA‐targeted polymers were employed to deliver a plasmid expressing the TRAIL protein, which induced death in cancer cells. These results indicate that FA‐conjugated aminoglycoside‐derived polymers are promising for targeted delivery of nucleic acids to cancer cells that overexpress folate receptors.

## 1 Introduction

Recent epidemiological studies performed by the National Cancer Institute's SEER program estimates approximately 250,000 new cases for breast cancer and 75,000 new cases for bladder cancer in the year 2016, resulting in the death of 16 and 20% of cases, respectively.^1^ Conventional chemotherapies face significant challenges for treating advanced cancers due to limitations associated with drug resistance, low bioavailability, and non‐specific activity, all of which can lower efficacy and/or lead to various side‐effects during treatment.

Folic acid (FA), also known as Vitamin B9, is an essential nutrient for humans. The uptake of FA is facilitated by two different mechanisms. The folate transporter protein called SLC19A1 facilitates endocytosis of a reduced form of folate, and is hence known as the reduced folate carrier (RFC1).^2^ Second, the high‐affinity folate receptor (FR) recognizes FA and facilitates internalization of the oxidized form by means of receptor‐mediated endocytosis. Following entry via FR‐mediated endocytosis, FA is then released into the cytosol, in a process thought to be mediated by endosomal acidification.^3^, ^4^, ^5^

Targeted delivery has the potential to increase retention of small‐molecule drugs and nucleic acids in tumors, which can result in enhanced efficacies for cancer cell ablation. The FR is over‐expressed in ovarian, non‐small cell lung cancer,^6^, ^7^, ^8^, ^9^, ^10^ and triple‐negative breast cancers,^11^ and has therefore been employed to target therapeutic cargo to tumors. The receptor was also shown to be overexpressed in bladder cancer, although to a modest extent in some cases.^12^, ^13^, ^14^, ^15^, ^16^ Lower expression levels of the receptor on non‐malignant cells offer a window for selective destruction of cancer cells.^16^ FR‐mediated endocytosis has therefore been employed for delivering small molecules and nucleic acids to cancer cells that over‐express the receptor.^17^, ^18^, ^19^, ^20^, ^21^, ^22^, ^23^, ^24^, ^25^

Aminoglycosides are a group of antibiotics which are used in the treatment of various bacterial infections,^26^, ^27^ although their use is somewhat limited because of concerns regarding nephrotoxicity. These molecules typically contain three to five sugar moieties, each of which contain multiple amines and/or hydroxyls. Aminoglycosides demonstrate anti‐bacterial activity through selective binding to the bacterial ribosomal RNA regions.^28^, ^29^ In addition, aminoglycosides have also been shown to have the capability to bind eukaryotic RNA and plasmid DNA (pDNA).^30^, ^31^, ^32^, ^33^ Given their chemical diversity, we employed aminoglycosides as starting materials for the generation of bioseparation ligands,^30^ DNA‐binding ligands,^30^, ^33^ and microbeads.^34^ Recently, we employed parallel synthesis and chemical informatics modeling for the generation and rapid identification of aminoglycoside‐derived polymers and lipopolymers for delivering transgenes to cancer cells.^35^, ^36^, ^37^, ^38^ These studies have led to the identification of polymers and lipopolymers that demonstrate higher efficacies for transgene expression than commonly used reagents (e.g., branched pEI).

**Scheme 1:**
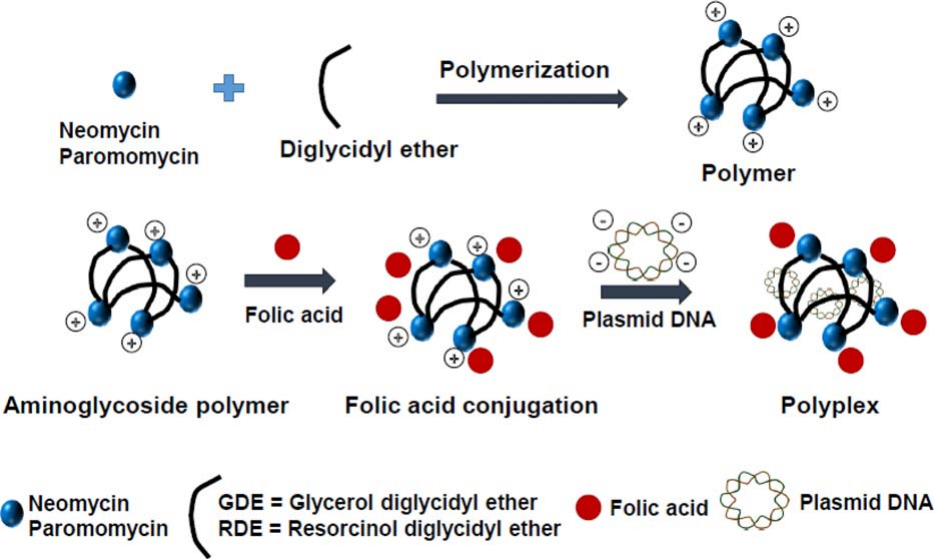
Pictorial representation of FA‐conjugated, aminoglycoside‐derived polymers for targeted delivery of plasmid DNA via folate receptors on cancer cells. Not to scale

In the present work, we have developed a targeted delivery approach with an eye toward facilitating the selective delivery of pDNA to cancer cells. In particular, we hypothesized that modification of efficacious aminoglycoside‐derived polymers with FA would be a powerful approach for delivery to cancer cells that overexpress the FR (Scheme 1). Four aminoglycoside‐derived polymers were derivatized with FA and their efficacy for transgene expression was evaluated in breast and bladder cancer cells. Effective polymers were then evaluated for delivering a plasmid that expresses the tumor necrosis factor‐alpha related apoptosis‐inducing ligand (TRAIL) protein, and the extent of cancer cell death was determined.

## 2 Experimental

### 2.1 Materials

FA, 1‐ethyl‐3‐(3‐dimethylaminopropyl) carbodiimide (EDCi), N‐hydroxysuccinimide (NHS), dimethyl sulfoxide (DMSO), glycerol diglycidyl ether (GDE), resorcinol diglycidyl ether (RDE), and diethyl ether were purchased from Sigma‐Aldrich, St. Louis, USA. The reagents were used without further purification. Aminoglycoside monomers, neomycin sulphate, and paromomycin sulphate were purchased from Gold Biotechnology, Inc., USA. Gel permeation chromatography (GPC) standards were purchased from American Polymer Standards Corporation. Lipofectamine‐3000 was a free sample from Invitrogen Life Technologies. The BCA protein assay kit was purchased from Thermo Scientific, Inc. (Rockford, IL). The pGL4.5, pGL3.0 control vectors, which encodes for modified firefly luciferase protein under the control of an SV promoter, and the Bright‐GloTM luciferase assay system were purchased from Promega Corporation (Madison, WI). Diethyl ether and acetone were purchased from VWR international LLC, USA suppliers and were used without further purification.

### 2.2 Synthesis of aminoglycoside‐derived polymers

The synthesis and characterization of aminoglycoside‐derived polymers (neomycin‐GDE, paromomycin‐GDE, neomycin‐RDE, and paromomycin‐RDE) were based on modifications made to methods described in our previous reports.^37^ Briefly, sulphate‐containing aminoglycosides neomycin and paromomycin were dissolved in nanopure water and passed through a Cl^‐^ ion exchange resin for 3 hr. The resulting sulphate‐free aminoglycosides were reacted with GDE or RDE in a 1:2.2 molar ratio in a solvent mixture of water and N,N‐dimethylformamide (DMF), (1.5:1 v/v) for 5 hr at 60°C. The reaction mixture was precipitated by using acetone, and was further purified by dialysis using a 3.5 kDa molecular weight cutoff (MWCO) dialysis membrane for 48 hr to remove unreacted aminoglycosides and diglycidylethers. The dialyzed material was lyophilized to obtain the purified polymers. Neomycin‐GDE, paromomycin‐GDE, neomycin‐RDE, and paromomycin‐RDE polymers are abbrevated as NG, PG, NR, and PR, respectively. These untargeted polymers and are also called parental polymers collectively in subsequent usage.

### 2.3 Synthesis and structural characterization of FA‐conjugated aminoglycoside‐derived polymers

NG, PG, NR, and PR aminoglycoside‐derived polymers were further derivatized with FA as shown in Scheme 1. The polymers (100 mg, 1 molar eq) were added to a 50 mL round bottomed flask and dissolved in 2 mL DMSO. FA (2 molar eq) was dissolved in 2 mL of DMSO in a different 50 mL round bottom flask. EDCi (2.2 molar eq), NHS (2.2 molar eq) were added to this flask and the contents were stirred for 30 min at room temperature. A unique polymer solution (i.e., NG, PG, NR, or PR) in DMSO was added to this reaction mixture, and the contents were stirred for 48 h at room temperature. The FA‐conjugated polymer product was obtained following precipitation with diethyl ether. The resulting polymer product was dissolved in water and dialyzed against Nanopure water for 48 hr using a 3.5 kDa MWCO tubing. The dialyzed material was lyophilized to obtain the final FA‐conjugated polymer products, which were abbreviated as NGFA, PGFA, PRFA, or NRFA (FA = folic acid).

Structural information on FA‐conjugated polymers dissolved in DMSO‐d_6_ was obtained using ^1^H NMR spectroscopy (Varian 500 instrument operating at 500 MHz in the Fourier transform mode); tetramethyl silane was employed as an internal reference. FT‐IR spectra were recorded with a Bruker IFS 66V/S 32 mm instrument using a round cell window (KBr), to further confirm FA conjugation onto parental aminoglycoside‐derived polymers.

### 2.4 GPC for molecular weight measurement

GPC was used to measure the molecular weights of parental and FA‐conjugated polymers. The Waters 1515 GPC system is equipped with an ultrahydrogel 250 column (Waters Corporation, MA) and a refractive index detector (Waters 2410). An aqueous solvent containing 0.1% trifloroacetic acid and 40% acetonitrile was used as the mobile phase. The mobile phase in the column was operated at a flow rate of 0.5 mL/min, and the column temperature was set at 35°C. Poly(2‐vinylpyridine) standards (MWs: 3,300, 7,600, 12,800, 35,000, and 70,000 Da) were employed for molecular weight (MW) calibration. All chromatograms were analyzed using the Waters Millennium 32 GPC software. To further investigate the MW range, the parental polymers were first dialyzed through a 3.5 kDa MWCO membrane to remove any unreacted monomers. Following this first step for 48 hr, the retentate was dialyzed through a 10 kDa MWCO membrane. Samples of retentates from the 3.5 and 10 kDa MWCO dialysis membranes were subjected to ninhydrin analysis which reports for reactive amine content. A calibration using glycine as a standard was used to determine the amine content of polymers, and the relative amounts of amines left behind in the retentates were compared to determine the MW range of the synthesized polymers.

### 2.5 Preparation of polymer‐pDNA complexes (polyplexes)

Stock solutions of parental (untargeted) and FA‐conjugated (targeted) polymers were prepared at a concentration of 2 mg/mL in 1× PBS (pH 7.4). These solutions were filtered through 0.2 µm filters before use. pDNA stock solutions were prepared in EB buffer (Qiagen, Germany) at a concentration of 50 ng/µL. For transgene expression studies, a total of 75 ng pDNA were complexed with varying amounts of polymers in 1X PBS buffer for 20 min, resulting in polyplexes at different polymer:pDNA weight ratios (w/w) ranging from 1:1 to 100:1.

### 2.6 Hydrodynamic diameters and zeta potential values of polyplexes

Hydrodynamic diameters and zeta potentials for all polymer:pDNA complexes (polyplexes) were determined using a Zetasizer Nanosystems Nano‐ZS instrument (Malvern Instruments, Mission Viejo, CA). Polyplexes were formed by complexing parental and FA‐conjugated polymers at optimal polymer:pDNA weight ratios of 5:1 to 25:1 for 20 min; these were determined as the optimal ratios from transgene expression studies. Polyplex volumes of 50 µL at different weight ratios in 1× PBS (i.e., 150 mM salt concentration, pH 7.4) were used; the amount of pDNA was kept constant at 75 ng (4 µL, 25 ng/µL in EB buffer) in all experiments. Zeta potential and hydrodynamic diameter measurements were carried out in triplicate.

### 2.7 Cell culture

MDA‐MB‐231 human triple‐negative breast cancer cells, and UMUC3 human bladder cancer cells were procured from the American Type Cell Culture (Manassas, VA). Cells were cultured in DMEM media supplemented with 10% v/v fetal bovine serum (FBS) and 1% penicillin‐streptomycin (10,000 units/mL) solution at 37°C under an atmosphere of air (95%) and carbon dioxide (5%) in an incubator. At approximately 80% confluence, cells were trypsinized with 0.25% trypsin‐EDTA and seeded at a density of 10,000 cells/well in 96‐well plates (Corning, Corning, NY, USA) for all cell viability experiments.

### 2.8 Transgene (luciferase) expression in cancer cells following pDNA delivery using FA‐conjugated polymers

FA‐conjugated polymers and parental polymers were evaluated for their transgene expression efficacies by delivering the pGL4.5 and pGL3.0 control vectors (Promega Corp., Madison, WI), which encode for the modified firefly luciferase protein, to MDA‐MB‐231 and UMUC3 cancer cells. The pGL4.5 and pGL3.0 pDNA were prepared using methods described previously.^28^ pDNA concentration and purity were determined using a NanoDrop Spectrophotometer (ND‐1000; NanoDrop Technologies) by measuring absorbance at 260 and 280 nm.

Cells were seeded at a density of 10,000 per well in a 96‐well plate and allowed to attach for 18–24 hr. Polymer: pGL4.5 plasmid weight ratios of 5:1, 10:1, 15:1, 20:1, and 25:1 were investigated in transgene (luciferase) expression studies; weight ratios of 20:1 and 25:1 polymer:pDNA were used in case of the pGL3.0 plasmid. All transfections were carried out in presence of media containing 10% FBS and 75 ng pDNA were used/well. After 48 hr of incubation with the polyplexes, cells were lysed and luciferase protein expression was determined as relative luminescence units (RLU) using the Bright GloTM Luciferase assay kit (Promega), in concert with a plate reader (Bio‐Tek Synergy 2). Total protein content was also assayed from the cell lysates using the BCA Protein Assay kit (Pierce, Rockford, IL, USA). RLU values were normalized by the protein content to yield “*RLU/mg protein*” values, which were employed for comparing different FA‐conjugated polymers with their respective parent polymers as well as Lipofectamine‐3000, which was used as a transfection standard in both UMUC3 and MDA‐MB‐231 cells using pGL3.0 plasmid for 48 hr of incubation according to vendor's protocols. Briefly, six different Lipofectamine‐3000 concentrations, 0.01, 0.05, 0.15, 0.3, 0.5, and 1.0 µL/well and P3000 reagent at concentrations 0.2 and 0.5 µL/well per 75 ng of DNA were used in these studies.

### 2.9 Polymer‐mediated enhanced green fluorescent protein transgene expression

All parental and FA‐conjugated polymers were employed to deliver a plasmid pEF‐enhanced green fluorescent protein (EGFP) which expresses EGFP to MDA‐MB‐231 and UMUC3 cells, using methods described for pGL4.5 plasmid above; the EGFP expression was under the control of an elongation factor 1α (EF1‐α) promoter. EGFP expression was visualized using an inverted fluorescence microscope (Zeiss, AxioVision D) in all cases.

### 2.10 Competitive inhibition of transgene expression with soluble FA in media

Cells were treated with free FA prior to the delivery of polyplexes as a competitive inhibition assay, to investigate the role of FR in transgene expression efficacy. MDA‐MB‐231 and UMUC3 cells were pre‐treated with 5 mM free FA^39^, ^40^, ^41^ in media for 6 hr, removed media and washed with 1 X PBS, followed by treatment with FA‐conjugated polymers and unconjugated parental polymers at an optimal polymer: pGL4.5 weight ratio of 25:1. After 48 hr, cells were investigated for their transgene (luciferase) expression efficacies in the presence of serum. As before, Lipofectamine‐3000 was used as a standard for comparison. The concentration of FA added to the media was significantly higher than that originally present in the media formulation from the vendor (FA in media = 9 µM).

### 2.11 Immunostaining for folate receptor alpha

Expression of FR‐α in UMUC3 cells was visualized using immunostaining, and T24 human bladder cancer cells were used as the positive control since expression of the FR is known in these cells^42^, ^43^; in addition, the expression of FR‐α in MDA‐MB‐231 cells is well established.^44^, ^45^ 300,000 UMUC3 or T24 cells/well were seeded onto cover slips and incubated for 24 hr and stained using 1:100 dilution of primary antibody against FR‐α (Mouse API3005AA, Biocare Medical) at room temperature followed by incubation with a 1:200 dilution of MACH 4 anti‐mouse probe (Rabbit UP534G, Biocare Medical) at room temperature for 1h. The cells were then incubated with an FITC‐tagged anti‐rabbit antibody using 1:400 dilution (Goat Alexa Fluor‐488, ThermoFisher Scientific) for 1 hr. The cells were twice washed with 1× PBS containing 2% FBS for 15 min before and after each antibody incubation step and visualized using a Leica TCS SP5 AOBS Spectral Confocal Microscopy System using 10×, 20×, and 40× (oil immersion) objectives; cells were excited at 488 nm and emission wavelengths of 530 nm were recorded. The same procedure was followed without the addition of the primary antibody as a negative control in these studies.

### 2.12 Cytotoxicity of polyplexes

The MTT ((3‐(4,5‐Dimethylthiazol‐2‐yl)−2,5‐diphenyltetrazolium bromide)) cell viability assay is a metabolic assay used to determine cell proliferation, and can be employed as an indirect reporter for cell viability. The MTT assay was employed for determining the cytotoxicity of polymer:pDNA complexes (weight ratios 5:1 to 25:1) in MDA‐MB‐231 and UMUC3 cells. Cell seeding and polyplex formation procedures were similar to those described in section 2E. Untreated control wells were treated only with media (i.e., no polymer). After 48 hr, 10 µL of the MTT reagent were added to the cells and they were incubated for 3 hr at 37°C following which, 50 µL methanol:dimethylsulfoxide (1:1) were added and incubated at room temperature for 30 min. After incubation, contents in the wells were thoroughly mixed, and absorbance at 570 nm was determined using a plate reader (Bio‐Tek Synergy 2). The relative cell viability (%) was calculated from [ab]test/[ab]control × 100%, where [ab]test and [ab]control are the absorbance values of the wells with polymers and control wells without polymers, respectively. For each sample, the final absorbance/cell viability was reported as the average of values measured from three wells in parallel.

### 2.13 Construction of the pEF‐TRAIL plasmid

To construct the pEF‐TRAIL plasmid, full‐length human TRAIL was first PCR‐amplified from the pEGFP‐TRAIL plasmid, kindly provided by Prof. Christina Voelkel Johnson and described here.^46^ Amplification was carried out using custom DNA oligonucleotides synthesized by Integrated DNA Technologies (Coralville, IA, USA) and a BioRad iCycler system with Q5 High Fidelity DNA Polymerase (New England Biolabs (NEB), Ipswich, MA, USA) according to manufacturer protocols. Amplified TRAIL DNA was incubated with ProteinaseK (NEB) according to manufacturer protocols, and subsequently purified using the Zymo Research DNA Clean & Concentrator MiniPrep (Zymo Research, Irvine, CA, USA) according to manufacturer protocols. Purified TRAIL DNA and pEF‐GFP (purchased from Addgene, plasmid # 11154), which contains the EGFP gene under the control of the EF1α promoter, were individually digested with EcoRI‐HF and NotI‐HF (NEB) according to manufacturer protocols; this step removed EGFP from the pEF‐GFP plasmid. Digested DNA fragments were gel‐purified using the Zymoclean Gel DNA Recovery MiniPrep (Zymo Research). Purified digested TRAIL and pEF‐GFP DNA were ligated with T4 DNA Ligase (NEB) according to manufacturer protocols, resulting in the pEF‐TRAIL plasmid with the EF1α promoter driving expression of TRAIL. Ligation reactions were transformed into chemically competent E. coli NEB10‐beta (NEB) and selected for on LB solid agar plates supplemented with 100 mg/L ampicillin. The resultant transformant pool was screened for correct clones using colony PCR, restriction digest mapping, and confirmed by DNA sequencing. Custom primer and pEF‐TRAIL DNA sequences are provided in the supplemental material (Figure S1**)**.

### 2.14 Delivery of pEF‐TRAIL to cancer cells using FA‐conjugated aminoglycoside‐derived polymers

We determined the loss of viability in MDA‐MB‐231 and UMUC3 cells following delivery of the pEF‐TRAIL plasmid using NR, PR, NRFA and PRFA polymers. Two optimal polymer:pDNA weight ratios, 15:1 and 25:1, were employed in these experiments. The pEF‐GFP expression vector, which expresses EGFP, was employed as a control in the study since it expresses EGFP which should not induce death in cancer cells. Polyplex formation and other experimental procedures employed in these studies were similar to that described previously in this section.

### 2.15 Western blotting

Expression of TRAIL in UMUC3 cells following delivery with FA‐conjugated and unconjugated polymers was determined using Western blots. UMUC3 cells were seeded at a density of 300,000 cells/well in a 6‐well plate and treated with NRFA PRFA, NR, and PR polymers complexed with 500 ng pEF‐TRAIL plasmid/well or 500 ng pEF‐GFP plasmid/well for 48 hr. These conditions were different from those in transfection studies above to facilitate the collection of sufficient protein for Western blotting. Protein (30 µg) was separated on 4–12% Bis/Tris NuPage gels (Invitrogen) and transferred to nitrocellulose for 2 hr at 30 V. Membranes were blocked using 5% milk in TBS‐Tween for a minimum of 1 hr prior to incubation with primary antibody (TRAIL, 1:1,000, Cell Signaling Technology; #3219 and anti‐Actin, 1:4,000, Sigma A2066). Following three washes with TBS‐Tween, membranes were incubated with HRP‐conjugated secondary anti‐rabbit antibody (1:10,000 dilution, SantaCruz Biotechnology, Inc.) for 1.5 hr at room temperature in TBS‐Tween. Membranes were washed three times in TBS‐Tween followed by detection of the secondary conjugates with Immobilon Western Chemiluminescent Solution (Millipore).

### 2.16 Statistical analyses

All cell‐based experiments were carried out in triplicate, and the results are expressed as mean ± one standard deviation using GraphPad Prism 7.0 (GraphPad Software, Inc.). The Student's *t*‐test was used to assess statistical significance of difference between group means; *p*‐values <.05, with respect to corresponding observations with Lipofectamine‐3000, are considered statistically significant.

## 3 Results

### 3.1 Synthesis and characterization of FA‐conjugated aminoglycoside‐derived polymers

FA‐conjugated polymers were synthesized as illustrated in Scheme S1 (Supporting Information section). The amine groups present in the parent aminoglycoside‐derived polymers NG, PG, NR, and PR were reacted with the carboxylic group in FA using a coupling reagent EDCi and a base NHS in DMSO, resulting in the formation of the product. Figure S2 (Supporting Information section) shows the ^1^H NMR spectrum of all parent polymers (NG, PG, NR, and PR), and Figure S3 (Supporting Information section) shows the ^1^H NMR spectrum of the respective FA‐conjugated polymers. Characteristic peaks for FA observed in the region 4–7.5 ppm in FA‐conjugated polymers were absent in parental polymers, and taken to indicate formation of FA‐conjugated polymers. Peaks in the 7–8.5 ppm range correspond to hydrogen atoms in the aromatic group of FA, peaks at 2–3.5 ppm, correspond to aliphatic chains, and those at 5.5 ppm correspond to the –O–CH–O group of the aminoglycoside backbone of the polymer. Further, using ^1^H NMR spectrum, we also determined the extent of modification to estimate the molar ratio of FA to parental polymer in NGFA, PGFA, NRFA, and PRFA. It was found that approximately one molecule of FA was conjugated to every molecule of the parental polymer (Table S1, Supporting Information section). FA conjugation to the parent polymers was further confirmed by FT‐IR spectroscopy (Figure S4, Supporting Information section). Peaks at ∼3,350 and ∼2,895 cm^−1^, corresponding to the hydroxyl and primary amino groups, respectively, can be seen in the spectra for the parental polymers. Characteristic peaks for FA observed at ∼3,100 and ∼3,475 cm^−1^ are indicative of conjugation of FA to the parental polymer.

GPC analysis indicated that parental polymers and FA‐conjugated polymers typically exhibited average molecular weights in the range of 3.2–5.3 kDa (Table 1). FA‐conjugated polymers showed a modest increase in their molecular weights, which is along expected lines. The polydispersity indices of FA‐conjugated polymers and parental polymers ranged from 1.1–1.3 (Table 1), indicating that these molecular species are relatively uniform in nature. Ninhydrin analysis (amine content) of retentate samples in the 3.5 kDa and 10 kDa membranes indicated that most polymer was retained in the 3.5 kDa MWCO membrane but very little was left behind in the retentate following dialysis through the 10 kDa MWCO membrane (Table S2, Supporting Information section). These results serve to further confirm molecular weights determined using GPC (Table 1).

**Table 1:** Number‐averaged (*M*_n_) and weight‐averaged (*M*_w_) molecular weights, listed in Daltons (Da), and polydispersity index (PDI) of polymers as determined using gel‐permeation chromatography (GPC)

No.	Polymer	*M*_n_	*M*_w_	PDI
1	NG	3,062	3,846	1.18
2	NGFA	3,274	4,139	1.01
3	PG	4,667	4,889	1.21
4	PGFA	5,325	5,752	1.02
5	PR	3,060	3,428	1.12
6	PRFA	3,369	3,970	1.22
7	NR	3,451	3,965	1.25
8	NRFA	3,821	4,357	1.15

### 3.2 Transgene expression efficacy and cytotoxicity of FA‐conjugated polymers

**Figure 1:**
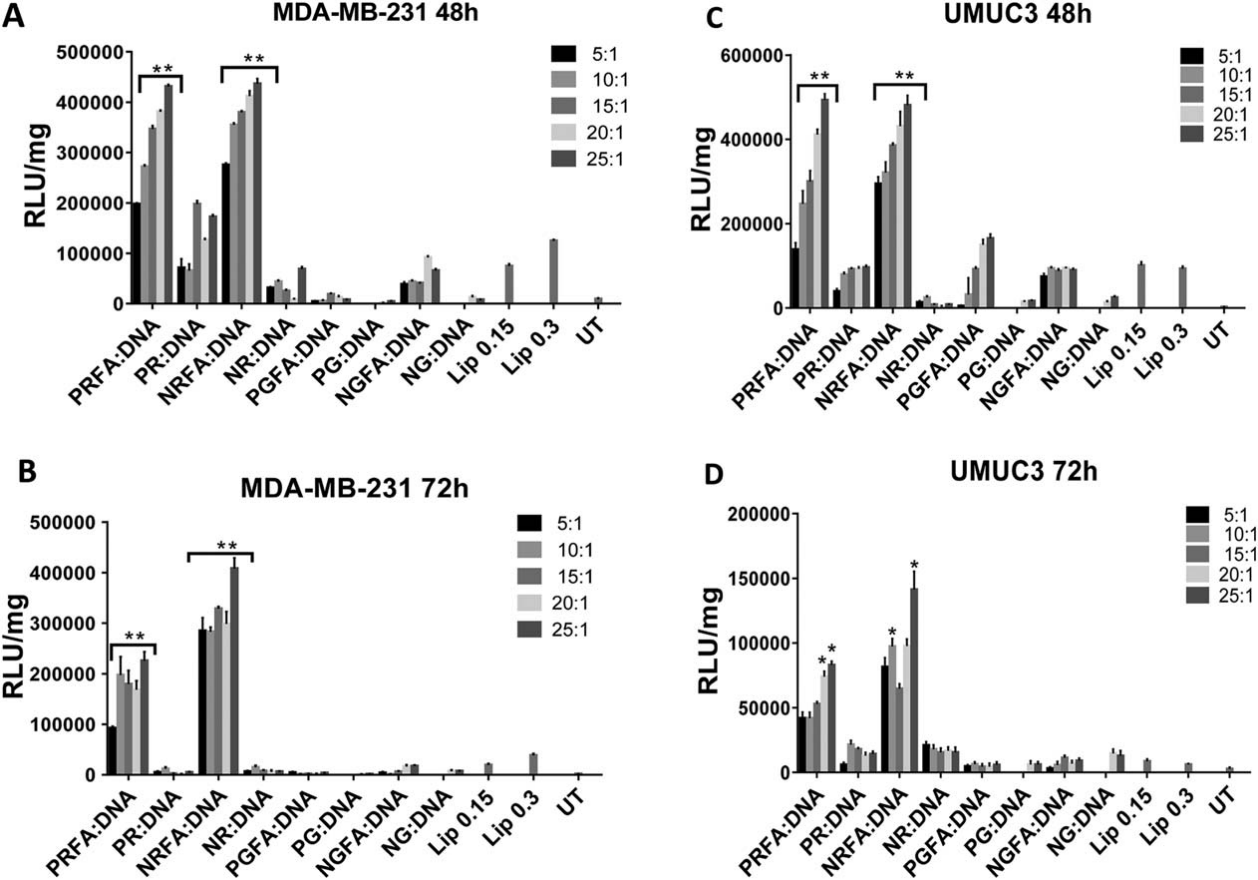
Luciferase transgene expression (RLU/mg) following delivery of the pGL4.5 plasmid using FA*‐*conjugated polymers, their corresponding parental polymers and Lipofectamine‐3000 (Lip) in (A) MDA‐MB‐231 (48 hr), (B) MDA‐MB‐231 (72 hr), (C) UMUC3 (48 hr) and (D) UMUC3 (72 hr) cells. Transgene expression was determined for polymer: pGL4.5 DNA weight ratios from 5:1 to 25:1. * = *p*‐value <.05; ** = *p*‐value <.01 using Student's *t*‐test; *p*‐values were obtained by comparing RLU/mg values of each FA*‐*conjugated polymer or the parental polymer with Lipofectamine‐3000 (Lip) under corresponding conditions. UT: untreated control. Data represent mean ± one standard deviation of three independent experiments (*n* = 3)

FA‐conjugated and parental polymers, at polymer:pDNA weight ratios ranging from 5:1 to 25:1, were employed for delivering the pGL4.5 and pGL3.0 plasmid to MDA‐MB‐231 human triple‐negative breast cancer and UMUC3 human bladder cancer cells. Luciferase expression efficacies of FA‐conjugated polymers and parental aminoglycoside polymers were compared to those observed with Lipofectamine‐3000 using RLU/mg values (described in the Experimental section) as shown in Figure 1A and B and Figure S5, Supporting Information section (for luciferase expression with pGL3.0 plasmid). Dose response studies indicated that higher concentrations (0.5 and 1.0 µL/well) of Lipofectamine‐3000 resulted in significant loss of cell viability (not shown) and lower efficacies of transgene expression (Figure S6 Supporting Information section). Based on these studies, optimal lipofectamine concentrations of 0.15 and 0.3 µL/well were used for comparison with parental and folate‐conjugated polymers.

NRFA and PRFA exhibited significantly higher luciferase protein expression levels than PGFA and NGFA and their corresponding parental polymers in both MDA‐MB‐231 and UMUC3 cells (Figure 1). Most polymers demonstrated relatively low efficacies of transgene expression at lower weight ratios of 5:1 to 15:1, likely due to negative zeta potential values observed under these conditions (Table 2); negative zeta potential values can reduce polyplex uptake by cells (discussed subsequently). Transgene expression efficacies were typically highest at weight ratios of 20:1 or 25:1, for both parental as well as FA‐conjugated polymers, although some variations were seen depending on polymer type. Note that the weight ratios used in these studies are lower than ones previously identified as optimal for the parental polymers, indicating higher efficacy of the folate‐targeted polymers. Luciferase expression levels observed with NRFA and PRFA polymers were ∼5–8 fold higher than those obtained using Lipofectamine‐3000, which is a common current standard for in vitro transgene delivery.

**Table 2:** Zeta potential values of polymer: pGL4.5 polyplexes of FA‐conjugated and parental polymers

Polymer	Polyplex zeta potential (mV)				
	5:1	10:1	15:1	20:1	25:1
NRFA	−1.2 ± 0.24	1.3 ± 1.27	3.4 ± 1.25	4.6 ± 1.79	6.4 ± 2.59
PRFA	−1.5 ± 1.56	0.9 ± 2.03	3.6 ± 2.56	4.1 ± 1.45	5.6 ± 2.04
NGFA	−3.8 ± 2.02	−1.2 ± 2.85	1.6 ± 1.98	2.3 ± 2.83	2.5 ± 2.73
PGFA	−3.2 ± 1.32	−2.4 ± 2.63	1.3 ± 1.81	1.7 ± 2.31	1.8 ± 2.89
NR	−4.3 ± 0.54	−1.9 ± 2.46	0.7 ± 2.01	1.2 ± 2.44	2.5 ± 2.65
PR	−3.3 ± 1.08	−1.8 ± 2.78	0.5 ± 2.08	1.5 ± 2.81	1.8 ± 2.74
NG	−4.8 ± 2.54	−3.8 ± 2.85	−1.3 ± 2.75	0.8 ± 3.14	1.1 ± 2.02
PG	−3.6 ± 2.46	−3.1 ± 3.9	−1.2 ± 2.39	0.2 ± 1.52	1.3 ± 2.87

**Figure 2:**
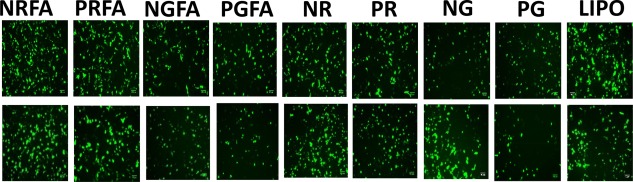
Expression of enhanced green fluorescent protein following delivery of EGFP plasmid to MDA‐MB‐231 (top row) and UMUC3 (bottom row) cancer cells as visualized using inverted fluorescence microscopy. The EF‐GFP plasmid was delivered using FA*‐*conjugated polymers, parental aminoglycoside‐derived polymers, and Lipofectamine‐3000 (0.3 µL/well); weight ratios that resulted in highest transgene expression efficacies, based on dose response studies (Figure 1), were used. Lipofectamine conditions were based on vendor's protocols. Representative images

FA‐conjugated polymers and parental polymers were also investigated for delivering the EGFP plasmid to investigate their efficacies with a different reporter protein; the EGFP plasmid expresses EGFP following transcription and translation. Fluorescence microscopy indicates that EGFP expression was higher when FA‐conjugated polymers were employed compared to the parental ones. Specifically, NRFA and PRFA polymers showed among the highest levels of EGFP transgene expression in both MDA‐MB‐231 and UMUC3 cells (Figure 2). These results indicate that different plasmids can be delivered using FA‐conjugated polymers leading to transgene expression.

Although cationic polymers are effective in facilitating high levels of transgene expression, their cytotoxicity can limit their application. For example, polyethyleneimine (pEI) can be an effective gene delivery polymer in certain cases, but its high toxicity is a limiting concern.^47^, ^48^, ^49^, ^50^ FA‐conjugated polymers and parental polymers demonstrated negligible toxicities even at relatively high concentrations employed (e.g., polymer:pDNA weight ratios of 25:1; Figure 3). The cytotoxicity of FA‐conjugated polymers was similar to that of unconjugated polymers.

**Figure 3:**
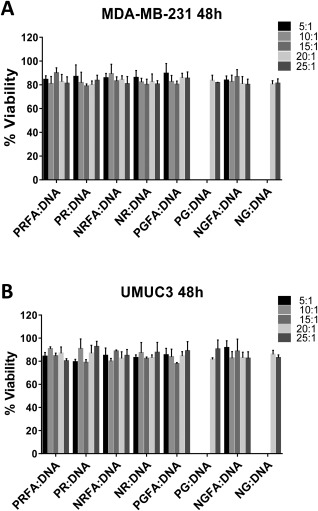
Cytotoxicity of polymer: pGL4.5 plasmid DNA complexes formed using FA*‐*conjugated and parent polymers at weight ratios ranging from 5:1 to 25:1 in (A) MDA‐MB‐231, and (B) UMUC3 cells. Data are presented as mean ± one standard deviation of three independent experiments (*n* = 3)

Taken together, our results indicate that FA‐conjugated polymers demonstrate higher transgene expression efficacies compared to their parental polymers, and their cytotoxicities are minimal under the conditions investigated.

### 3.3 Hydrodynamic diameter and zeta potential of polyplexes

**Figure 4:**
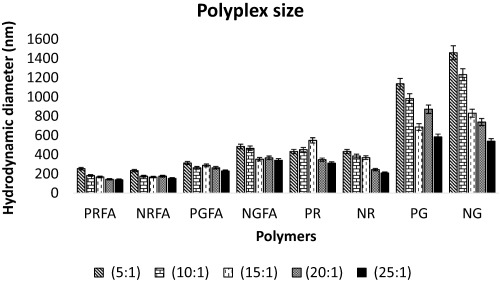
Hydrodynamic diameters (nm) of polymer: pGL4.5 plasmid polyplexes formed by FA‐conjugated polymers and their parent aminoglycoside polymers. The legend indicates different polymer:pDNA weight ratios ranging from 5:1 to 25:1. Each data point represents the mean ± one standard deviation of three independent experiments (*n* = 3)

Polyplexes less than 200 nm in hydrodynamic diameter are effectively internalized by cells compared to larger polyplexes and are therefore likely to result in higher levels of transgene expression.^51^, ^52^ The hydrodynamic diameter of polyplexes formed using FA‐conjugated polymers and parental polymers complexed with pGL4.5 pDNA at weight ratios ranging from 5:1 to 25:1 are shown in Figure 4. The sizes of polyplexes formed using NRFA and PRFA polymers were smaller than those formed using their corresponding parent polymers. NRFA and PRFA polymers, at weight ratios of 20:1 and 25:1, resulted in the formation of polyplexes with hydrodynamic diameters of approximately 140 nm, which, in part, can explain the higher levels of transgene expression seen in these conditions. These larger sizes of parental polymer (NG and PG) complexes indicate inefficient complexation between pDNA and parent polymers under these conditions, which is the likely cause of poor transgene expression efficacies under these conditions. However, sizes of the polyplexes formed by FA*‐*conjugated polymers (NRFA and PRFA) were smaller than those formed using the parental polymers (NR and PR) at weight ratios ranging from 5:1 to 25:1.

Zeta potential values of polymer:pDNA polyplexes are reflective of the surface charge of these complexes, and are known to influence interactions with anionic membranes of mammalian cells, facilitating their internalization. Polyplexes formed by complexing FA‐conjugated polymers with pGL4.5 pDNA demonstrated zeta‐potential values in the range of −4 to +6 mV across the different polymer:pDNA weight ratios evaluated (i.e., 5:1 to 25:1). Polyplexes formed with polymer:pDNA weight ratios <10:1 demonstrate near‐neutral surface charge; indeed negative zeta potential values were observed for polymer:pDNA weight ratio of 5:1. Negative zeta potential values can result in low uptake of polyplexes by cells^53^ and can partially explain the lower levels of luciferase expression seen under these conditions. Polyplexes formed with polymer:pDNA weight ratios >10:1 demonstrate modestly positive values of zeta potential, which is indicative of excess cationic surface charge on binding (Table 2). Positive zeta potential values can result in increased uptake of polyplexes by cells, leading to increased transgene expression levels, as seen under these conditions.

### 3.4 Transgene expression in presence of free FA

**Figure 5:**
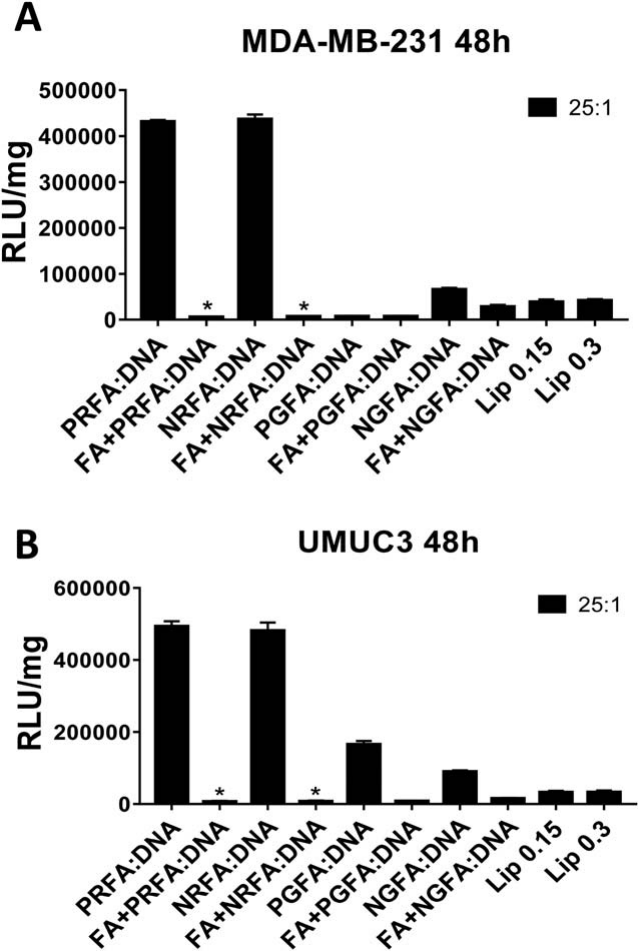
Transgene (luciferase) expression following FA‐conjugated polymer‐mediated delivery of pGL4.5 plasmid DNA after pre‐treatment of folic acid (5 mM concentration) to (A) MDA‐MB‐231, and (B) UMUC3 cancer cells. Transgene expression was evaluated at a weight ratio 25:1 of polymer:pDNA. Luciferase expression efficacies of all polymers were quantified in terms of relative luciferase units (RLU), and normalized to total protein content (mg), resulting in RLU/mg values. Luciferase expression efficacies of FA‐conjugated polymers were compared with that of FA‐conjugated polymers with folic acid pre‐treatment. Lipofectamine‐3000 is included as a control. * = *p*‐value <.01 using Student's *t*‐test; *p*‐values were obtained by comparing RLU/mg values of each FA*‐*conjugated polymer and the FA+ FA*‐*conjugated polymer

Expression of folate receptor alpha (FR‐α) in UMUC3 cells was verified using immunostaining (Figure S7 Supporting Information section); expression of FR‐α on MDA‐MB‐231 cells has been previously established.^44^, ^45^ Competitive inhibition studies are often employed to elucidate the role of a receptor in facilitating selective uptake of molecular cargo.^39^, ^40^, ^47^ We therefore pre‐treated cancer cells with free FA in media for 6 hr prior to delivery of polyplexes. Pre‐treatment with 5 mM FA resulted in near‐complete loss of the transgene expression efficacy of FA‐conjugated polymers (Figure 5), indicating that receptor‐mediated uptake played a role in polyplex uptake and resulting transgene expression.

### 3.5 Delivery of TRAIL‐expressing pDNA

**Figure 6:**
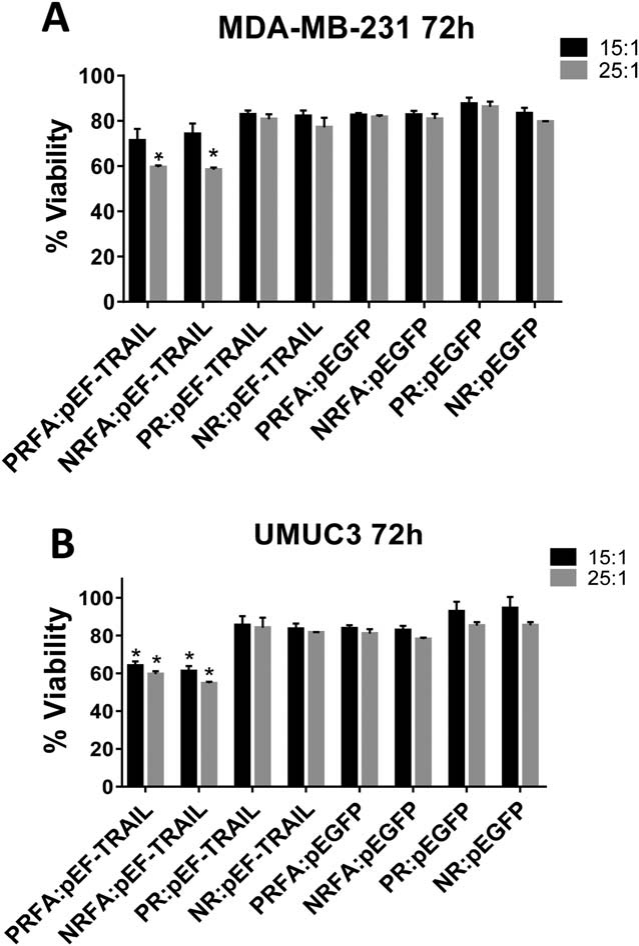
Cancer cell ablation efficacy following delivery of pEF‐TRAIL plasmid using different polymer:pDNA complexes at weight ratios 15:1 and 25:1 in (A) MDA‐MB‐231, and (B) UMUC3 cells. Data are presented as mean ± one standard deviation of three independent experiments (*n* = 3). * indicates *p*‐value <.05, when comparing FA‐conjugated polymers or their respective parental unconjugated polymers complexed with pEF‐TRAIL plasmid to those complexed with pEGFP plasmid

TRAIL is a protein that has been shown to induce programed cell death in cancer cells with minimal effect on normal cells, which makes it an attractive therapeutic for cancer diseases.^46^, ^54^, ^55^ TRAIL induces death in cancer cells by engaging the cellular apoptosis machinery following binding to death receptors DR4 and DR5.^55^ Folate‐conjugated, aminoglycoside‐derived polymers were employed to deliver a newly constructed plasmid designed to express the TRAIL protein. A pEGFP expression vector plasmid (pDNA) was employed as control. Western blots confirmed the expression of the TRAIL protein in UMUC3 cells following delivery of the pEF‐TRAIL plasmid using both parental and folate‐targeted NR and PR polymers (Figure S8, Supporting Information section). TRAIL expression, however, was not seen following delivery of the pEF‐GFP plasmid with encodes for EGFP. Cancer cell death induced by FA‐conjugated polymers and their corresponding parent polymers complexed with pEF‐TRAIL or EGFP plasmids was determined using the MTT assay at 72 h; although expression typically reaches maximal values at 48 hr (e.g., Figure 1), we determined viability at 72 to allow sufficient time for the expressed TRAIL protein to carry out its function. NRFA and PRFA polymers complexed with the pEF‐TRAIL showed a modest, but statistically significant decrease in MDA‐MB‐231 and UMUC3 cancer cell viability compared to polymers complexed with the EGFP plasmid (Figure 6); approximately ∼30–40% death of cancer cells was seen under these conditions.

## 4 Discussion

The FR is overexpressed on several cancer cell types including breast, ovarian, endometrial, brain, and bladder cancer.^16^, ^25^, ^42^ FA is a small molecule (441 Da) and is internalized in cells either by binding to FRs alpha, beta, and gamma (FOLR1‐3) or by a solute carrier protein SLC19A1 (RFC‐1) that can transport folate via a proton pump.^23^, ^56^ Targeting the FR using FA is an attractive strategy for delivering therapeutic cargo (e.g., small molecules and nucleic acids) specifically to cancer cells.^25^ Folate targeting has also been previously explored for delivering nucleic acids (antisense oligonucleotides, pDNA, or siRNA) using different macromolecular formulations,^57^, ^58^, ^59^, ^60^, ^61^, ^62^, ^63^, ^64^ and folate‐targeted lipids have also been investigated for delivering genes in vivo.^65^ In addition, folate‐targeted therapeutics have been investigated in clinical trials, which indicates the translational promise of this strategy.^66^ We have previously demonstrated that aminoglycoside‐derived polymers and lipopolymers demonstrated effective levels of transgene expression.^36^, ^37^, ^38^ Although aminoglycoside antibiotics can be nephrotoxic in their monomeric form, it is not clear if polymers derived from aminoglycosides demonstrate this activity. Given the high efficacy of these polymers, we hypothesized that conjugating ligands that can bind receptors on cancer cells can lead to targeted delivery vehicles. In this study, aminoglycoside‐derived polymers were conjugated with FA and the efficacy of transgene expression of these polymers was determined in MDA‐MB‐231 and UMUC3 cells.

Transgene expression in MDA‐MB‐231 and UMUC3 cells was significantly higher for FA‐conjugated paramomycin‐RDE (PRFA) and neomycin‐RDE (NRFA) polymers at different polymer: DNA ratios tested compared to lipofectamine‐3000, a commercially available transfection reagent. The transgene expression efficacy of these polymers was highest at a polymer:pDNA ratio of 25:1; with a 400–900% increase compared to the parental polymers under the conditions investigated. Competitive inhibition studies indicated that presence of free FA in media inhibited transgene expression, indicating a role for the FR in the gene delivery activity of the targeted polymers. Polyplexes with negative zeta potential values, observed at lower polymer:pDNA ratios, demonstrated lower transgene expression efficacies compared to those that demonstrated positive zeta potential values, seen in case of higher polymer content/polymer:pDNA ratios. At polyplex weight ratios of 25:1, the hydrodynamic diameters of NRFA and PRFA polyplexes were lower than 200 nm, which indicates efficient compaction that contributes to high levels of transgene expression.

TRAIL is a member of the Fas ligand family that can induce death selectively in cancer cells by engaging the death receptor machinery. Reports indicate that TRAIL induces minimal toxicity in normal tissues compared to cancer cells and as a result, TRAIL has been investigated in clinical trials either by itself or in combination with other drugs.^54^, ^67^, ^68^ Monoclonal antibodies, including mapatumumab, lexatumumab, conatumumab, tigatuzumab, and DAB4, that are agonistic to death receptors are currently in clinical trials for different solid and nonsolid malignancies.^54^, ^67^, ^68^ PRFA and NRFA polymer‐mediated delivery of a plasmid expressing the TRAIL protein resulted in death in a modest number of cancer cells. Although the level of cell death was not extensive, this is a promising result, since the efficacy of cancer ablation using TRAIL can be enhanced using chemotherapeutic drugs.^69^, ^70^, ^71^

## 5 Conclusions

In this study, we synthesized and characterized FA conjugated aminoglycoside‐derived polymers for delivering pDNA to cancer cells. Polyplexes formed using these targeted polymers were less than 200 nm in diameter and possessed a slightly positive surface charge. FA conjugated polymers demonstrated significantly higher levels of luciferase expression in cancer cells compared to their parental polymers; competitive inhibition of the FR inhibited transgene expression activity. Delivery of the TRAIL plasmid using these targeted polymers led to death in 30–40% cancer cells, which, although modest is promising since TRAIL can be synergized with other therapeutics resulting in significant loss of viability in cancer cells. Our results indicate that FA conjugated, aminoglycoside‐derived polymers may be promising vehicles for delivering nucleic acids and imaging agents to cancer cells that overexpress this receptor.

## Supporting information

Additional Supporting Information can be found online in the supporting information tab for this article.

Supporting Information
