# Supplementary material for: Folate receptor‐targeted aminoglycoside‐derived polymers for transgene expression in cancer cells
Source: Bioeng Transl Med. 2016 Oct 21;1(2):220–31. doi: 10.1002/btm2.10038 (PMC5675079; doi:10.1002/btm2.10038)
Supplement: Supplementary file 1 — Supporting Information [file BTM2-1-220-s001.doc]

**Supplementary information**

**Folate Receptor-targeted Aminoglycoside-derived Polymers for Transgene Expression in Cancer Cells**

**Sudhakar Godeshala^a^, Rajeshwar Nitiyanandan^b^, Brian Thompson**^a^**, Sheba Goklany^a^, David R. Nielsen**^a^**, and Kaushal Rege^a (*)^**

^a^Chemical Engineering

^b^Biological Design Program

Arizona State University, Tempe, AZ 85287-6106, USA.

^(*)^ To Whom All Correspondence is to be addressed

Kaushal Rege

Chemical Engineering

501 E. Tyler Mall, ECG 303

Arizona State University

Tempe, AZ 85287-6106

Email: [rege@asu.edu](mailto:kaushal.rege@asu.edu)

Phone: 480-727-8616

Fax: 480-727-9321

**Primer sequences**

TRAIL -- EcoRI - F

ATA GAA TTC GCC GCC ACC ATG GCT ATG ATG GAG GTC CAG GG

TRAIL -- NotI - R

ATT GCG GCC GCT TAG CCA ACT AAA AAG GCC CCA AAA AAA CTG

**Complete pEF-TRAIL sequence (*portions* *confirmed by sequencing*)**

gtcgacattgattattgactagatcatcgcgtgaggctccggtgcccgtcagtgggcagagcgcacatcgcccacagtccccgagaagttggggggaggggtcggcaattgaaccggtgcctagagaaggtggcgcggggtaaactgggaaagtgatgtcgtgtactggctccgcctttttcccgagggtgggggagaaccgtatataagtgcagtagtcgccgtgaacgttctttttcgcaacgggtttgccgccagaacacaggtaagtgccgtgtgtggttcccgcgggcctggcctctttacgggttatggcccttgcgtgccttgaattacttccacgcccctggctgcagtacgtgattcttgatcccgagcttcgggttggaagtgggtgggagagttcgaggccttgcgcttaaggagccccttcgcctcgtgcttgagttgaggcctggcctgggcgctggggccgccgcgtgcgaatctggtggcaccttcgcgcctgtctcgctgctttcgataagtctctagccatttaaaatttttgatgacctgctgcgacgctttttttctggcaagatagtcttgtaaatgcgggccaagatctgcacactggtatttcggtttttggggccgcgggcggcgacggggcccgtgcgtcccagcgcacatgttcggcgaggcggggcctgcgagcgcggccaccgagaatcggacgggggtagtctcaagctggccggcctgctctggtgcctggcctcgcgccgccgtgtatcgccccgccctgggcggcaaggctggcccggtcggcaccagttgcgtgagcggaaagatggccgcttcccggccctgctgcagggagctcaaaatggaggacgcggcgctcgggagagcgggcgggtgagtcacccacacaaaggaaaagggcctttccgtcctcagccgtcgcttcatgtgactccacggagtaccgggcgccgtccaggcacctcgattagttctcgagcttttggagtacgtcgtctttaggttggggggaggggttttatgcgatggagtttccccacactgagtgggtggagactgaagttaggccagcttggcacttgatgtaattctccttggaatttgccctttttgagtttggatcttggttcattctcaagcctcagacagtggttcaaagtttttttcttccatttcaggtgtcgtgaggaattcgccgccaccatggctatgatggaggtccaggggggacccagcctgggacagacctgcgtgctgatcgtgatcttcacagtgctcctgcagtctctctgtgtggctgtaacttacgtgtactttaccaacgagctgaagcagatgcaggacaagtactccaaaagtggcattgcttgtttcttaaaagaagatgacagttattgggaccccaatgacgaagagagtatgaacagcccctgctggcaagtcaagtggcaactccgtcagctcgttagaaagatgattttgagaacctctgaggaaaccatttctacagttcaagaaaagcaacaaaatatttctcccctagtgagagaaagaggtcctcagagagtagcagctcacataactgggaccagaggaagaagcaacacattgtcttctccaaactccaagaatgaaaaggctctgggccgcaaaataaactcctgggaatcatcaaggagtgggcattcattcctgagcaacttgcacttgaggaatggtgaactggtcatccatgaaaaagggttttactacatctattcccaaacatactttcgatttcaggaggaaataaaagaaaacacaaagaacgacaaacaaatggtccaatatatttacaaatacacaagttatcctgaccctatattgttgatgaaaagtgctagaaatagttgttggtctaaagatgcagaatatggactctattccatctatcaagggggaatatttgagcttaaggaaaatgacagaatttttgtttctgtaacaaatgagcacttgatagacatggaccatgaagccagtttttttggggcctttttagttggctaagcggccgcactcctcaggtgcaggctgcctatcagaaggtggtggctggtgtggccaatgccctggctcacaaataccactgagatctttttccctctgccaaaaattatggggacatcatgaagccccttgagcatctgacttctggctaataaaggaaatttattttcattgcaatagtgtgttggaattttttgtgtctctcactcggaaggacatatgggagggcaaatcatttaaaacatcagaatgagtatttggtttagagtttggcaacatatgccatatgctggctgccatgaacaaaggtggctataaagaggtcatcagtatatgaaacagccccctgctgtccattccttattccatagaaaagccttgacttgaggttagattttttttatattttgttttgtgttatttttttctttaacatccctaaaattttccttacatgttttactagccagatttttcctcctctcctgactactcccagtcatagctgtccctcttctcttatgaagatccctcgacctgcagcccaagcttggcgtaatcatggtcatagctgtttcctgtgtgaaattgttatccgctcacaattccacacaacatacgagccggaagcataaagtgtaaagcctggggtgcctaatgagtgagctaactcacattaattgcgttgcgctcactgcccgctttccagtcgggaaacctgtcgtgccagcggatccgcatctcaattagtcagcaaccatagtcccgcccctaactccgcccatcccgcccctaactccgcccagttccgcccattctccgccccatggctgactaattttttttatttatgcagaggccgaggccgcctcggcctctgagctattccagaagtagtgaggaggcttttttggaggcctaggcttttgcaaaaagctaacttgtttattgcagcttataatggttacaaataaagcaatagcatcacaaatttcacaaataaagcatttttttcactgcattctagttgtggtttgtccaaactcatcaatgtatcttatcatgtctggatccgctgcattaatgaatcggccaacgcgcggggagaggcggtttgcgtattgggcgctcttccgcttcctcgctcactgactcgctgcgctcggtcgttcggctgcggcgagcggtatcagctcactcaaaggcggtaatacggttatccacagaatcaggggataacgcaggaaagaacatgtgagcaaaaggccagcaaaaggccaggaaccgtaaaaaggccgcgttgctggcgtttttccataggctccgcccccctgacgagcatcacaaaaatcgacgctcaagtcagaggtggcgaaacccgacaggactataaagataccaggcgtttccccctggaagctccctcgtgcgctctcctgttccgaccctgccgcttaccggatacctgtccgcctttctcccttcgggaagcgtggcgctttctcaatgctcacgctgtaggtatctcagttcggtgtaggtcgttcgctccaagctgggctgtgtgcacgaaccccccgttcagcccgaccgctgcgccttatccggtaactatcgtcttgagtccaacccggtaagacacgacttatcgccactggcagcagccactggtaacaggattagcagagcgaggtatgtaggcggtgctacagagttcttgaagtggtggcctaactacggctacactagaaggacagtatttggtatctgcgctctgctgaagccagttaccttcggaaaaagagttggtagctcttgatccggcaaacaaaccaccgctggtagcggtggtttttttgtttgcaagcagcagattacgcgcagaaaaaaaggatctcaagaagatcctttgatcttttctacggggtctgacgctcagtggaacgaaaactcacgttaagggattttggtcatgagattatcaaaaaggatcttcacctagatccttttaaattaaaaatgaagttttaaatcaatctaaagtatatatgagtaaacttggtctgacagttaccaatgcttaatcagtgaggcacctatctcagcgatctgtctatttcgttcatccatagttgcctgactccccgtcgtgtagataactacgatacgggagggcttaccatctggccccagtgctgcaatgataccgcgagacccacgctcaccggctccagatttatcagcaataaaccagccagccggaagggccgagcgcagaagtggtcctgcaactttatccgcctccatccagtctattaattgttgccgggaagctagagtaagtagttcgccagttaatagtttgcgcaacgttgttgccattgctacaggcatcgtggtgtcacgctcgtcgtttggtatggcttcattcagctccggttcccaacgatcaaggcgagttacatgatcccccatgttgtgcaaaaaagcggttagctccttcggtcctccgatcgttgtcagaagtaagttggccgcagtgttatcactcatggttatggcagcactgcataattctcttactgtcatgccatccgtaagatgcttttctgtgactggtgagtactcaaccaagtcattctgagaatagtgtatgcggcgaccgagttgctcttgcccggcgtcaatacgggataataccgcgccacatagcagaactttaaaagtgctcatcattggaaaacgttcttcggggcgaaaactctcaaggatcttaccgctgttgagatccagttcgatgtaacccactcgtgcacccaactgatcttcagcatcttttactttcaccagcgtttctgggtgagcaaaaacaggaaggcaaaatgccgcaaaaaagggaataagggcgacacggaaatgttgaatactcatactcttcctttttcaatattattgaagcatttatcagggttattgtctcatgagcggatacatatttgaatgtatttagaaaaataaacaaataggggttccgcgcacatttccccgaaaagtgccacctg

**Figure S1.** Primer sequence and complete pEF-TRAIL DNA sequences.

**Scheme S1.** Schematic showing synthesis of the paromomycin/neomycin-resorcinol/glycerol diglycidyl ether parent polymers and synthesis of the folic acid conjugated polymers carried out in presence of EDCi/NHS. This illustration is meant to indicate the general reactions that take place and not the exact structure of the resulting product formed.


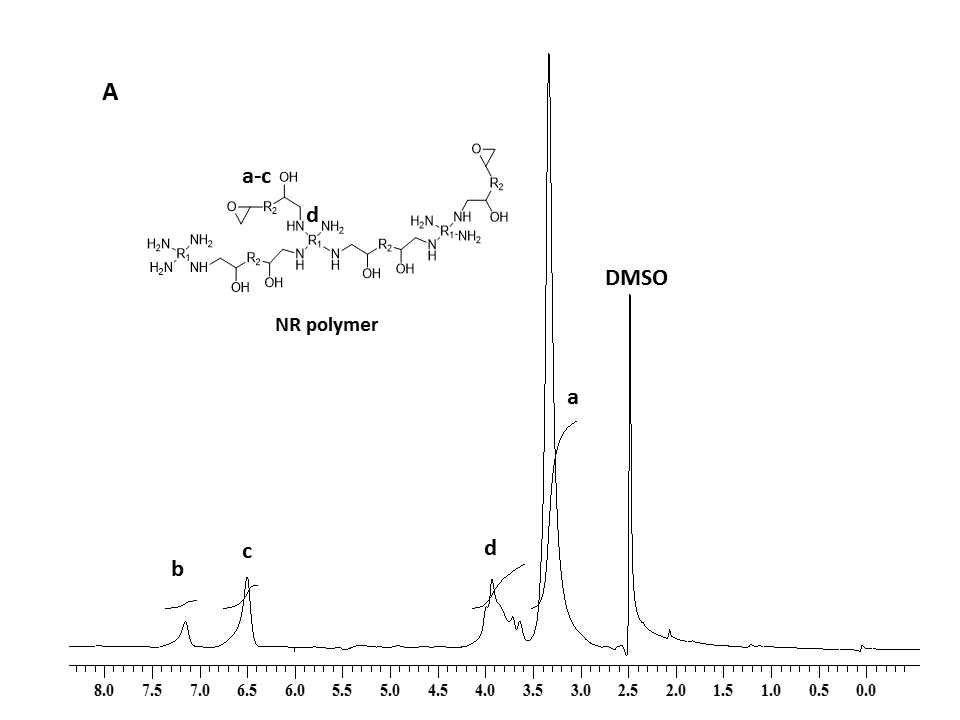


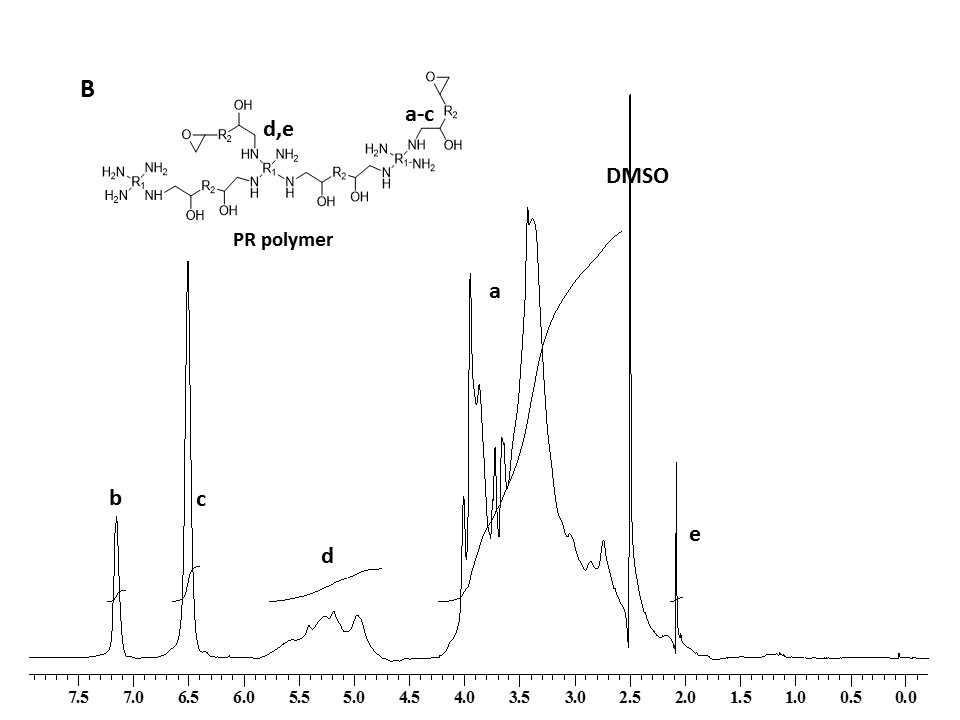


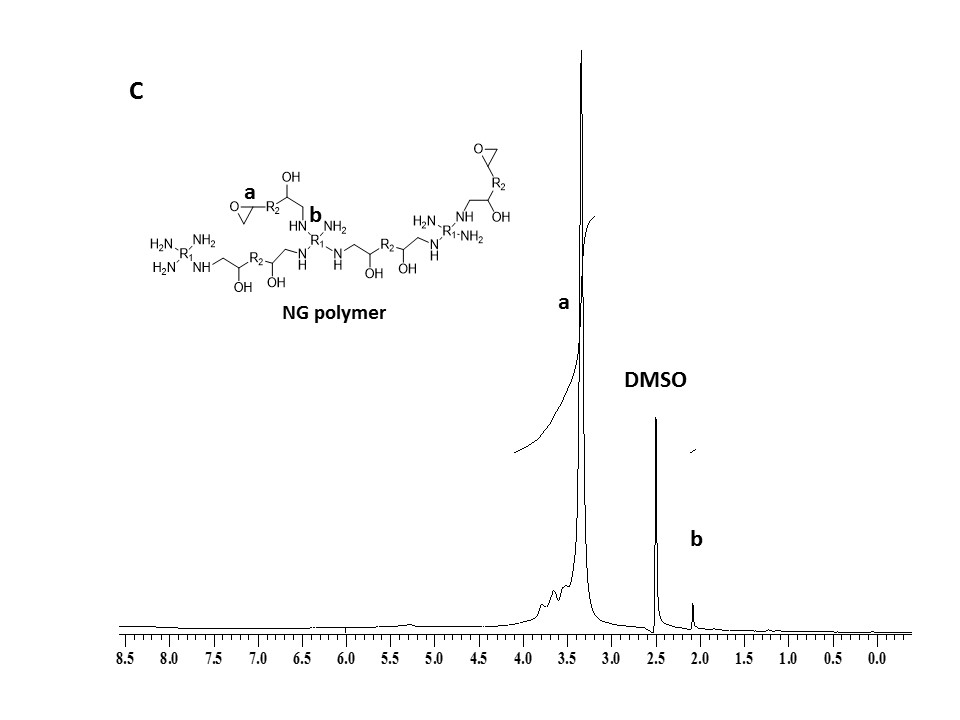


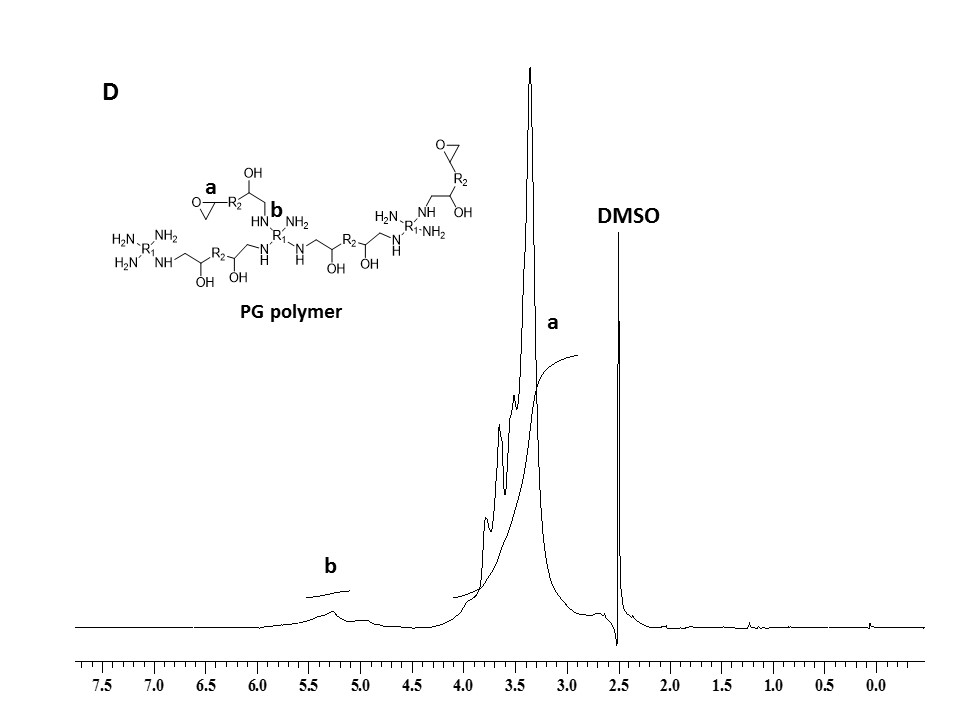


**Figure S2**. ^1^H-NMR spectra of parent polymers (A) NR, (B) PR, (C) NG, and (D) PG using DMSO-d_6_ as the solvent. Nomenclature of the different polymers is described in the Experimental section.


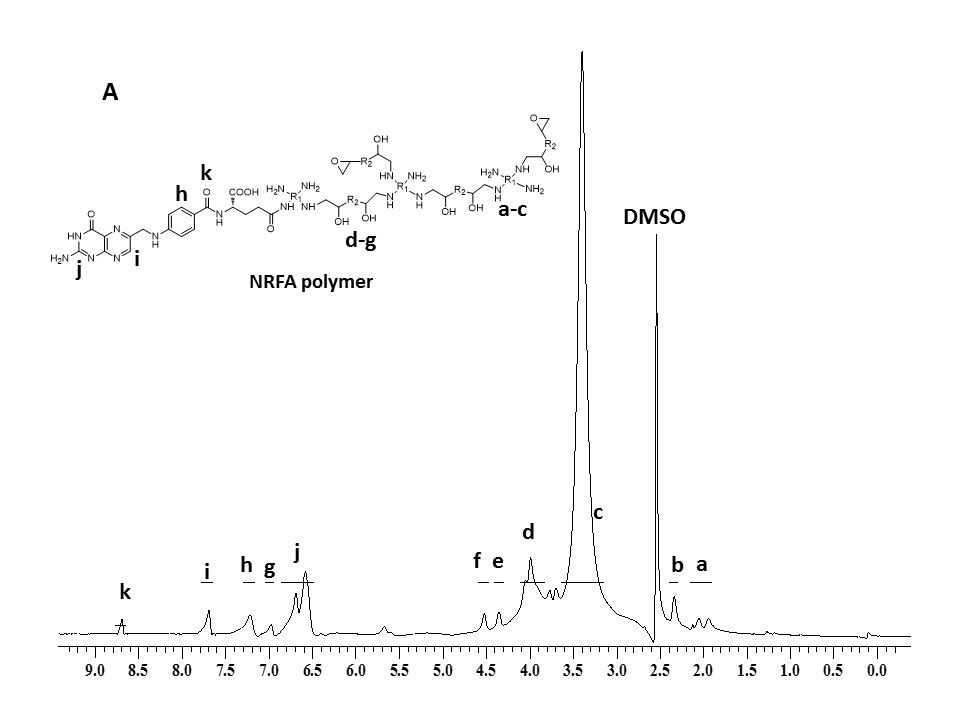


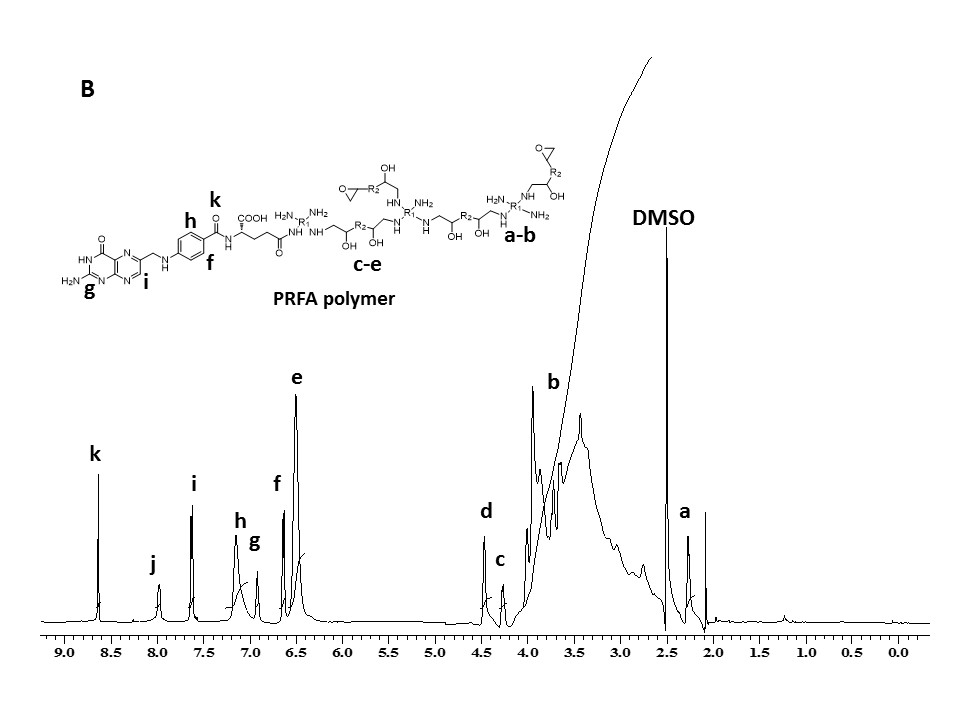


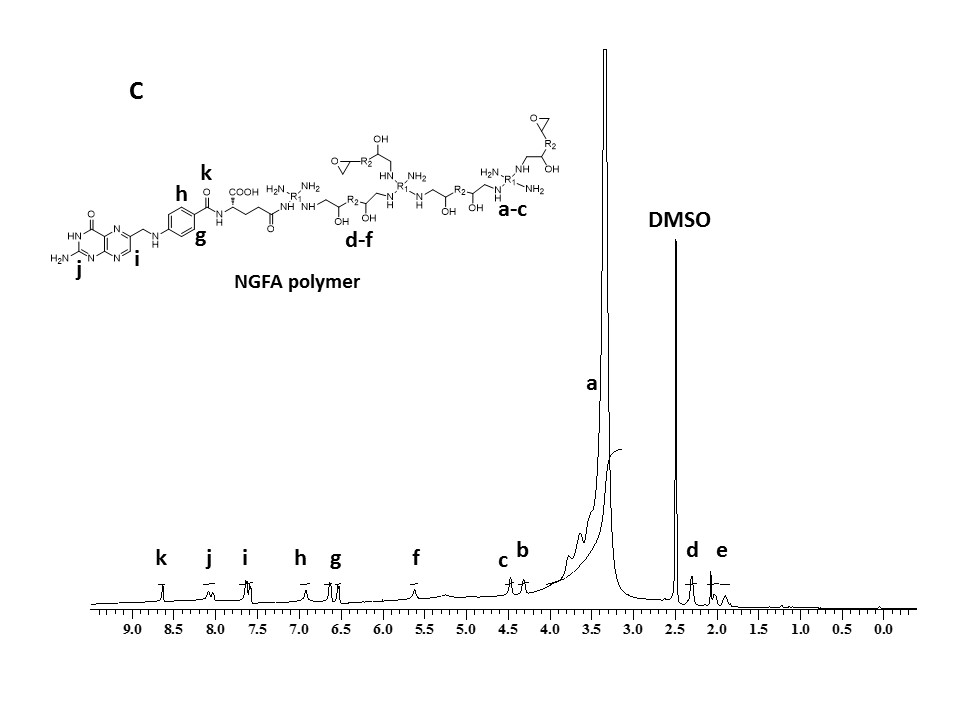


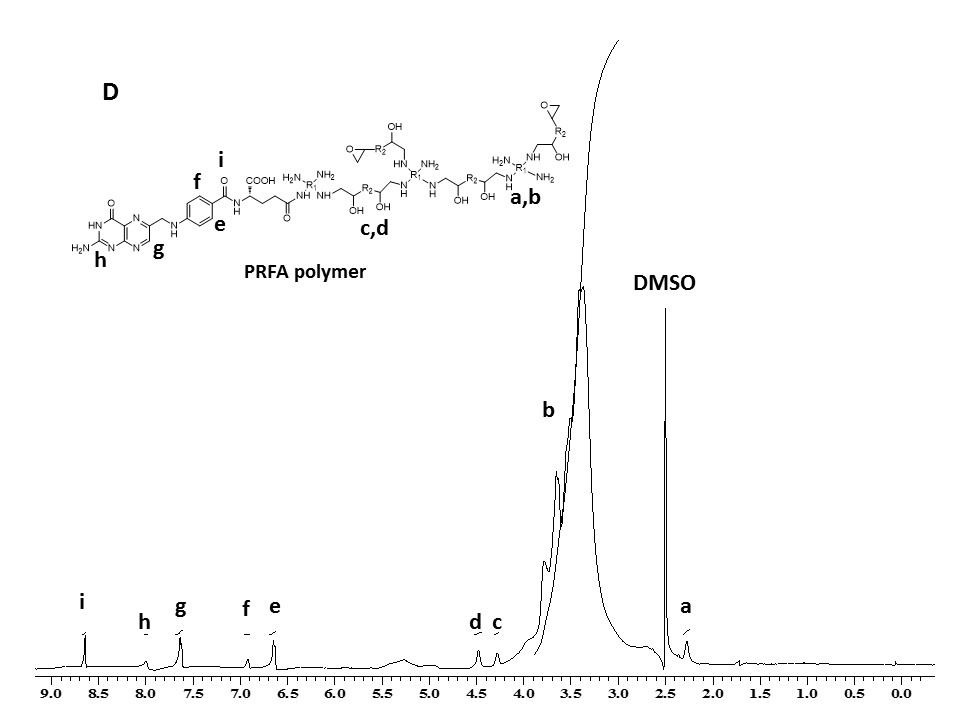


**Figure S3**. ^1^H-NMR spectra of folic acid conjugated polymers (A) NRFA, (B) PRFA, (C) NGFA, and (D) PGFA using DMSO-d_6_ as the solvent. Proton peaks corresponding to folic acid shown in blue circle. Nomenclature of the different polymers is described in the Experimental section.

**
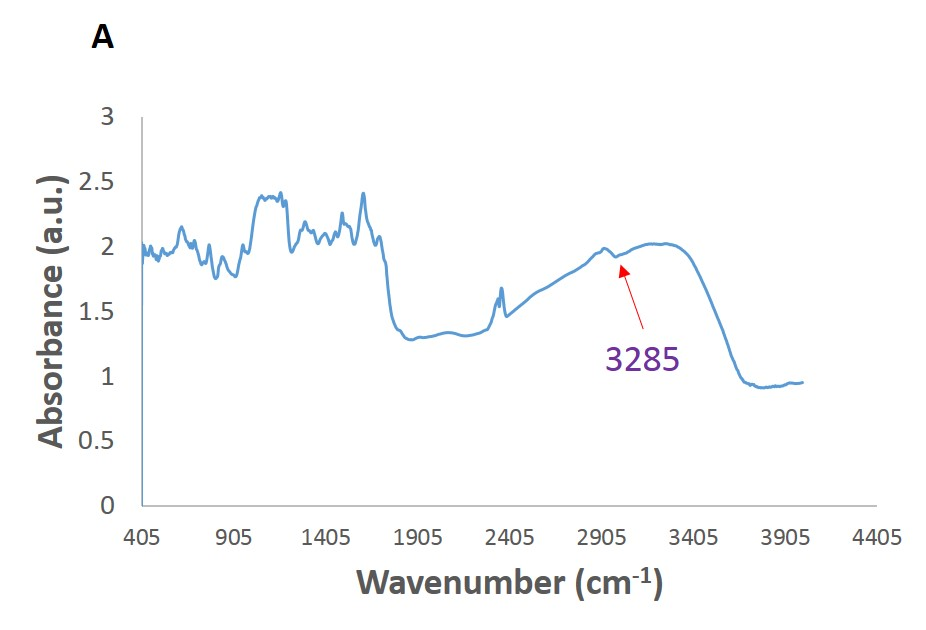
**

**
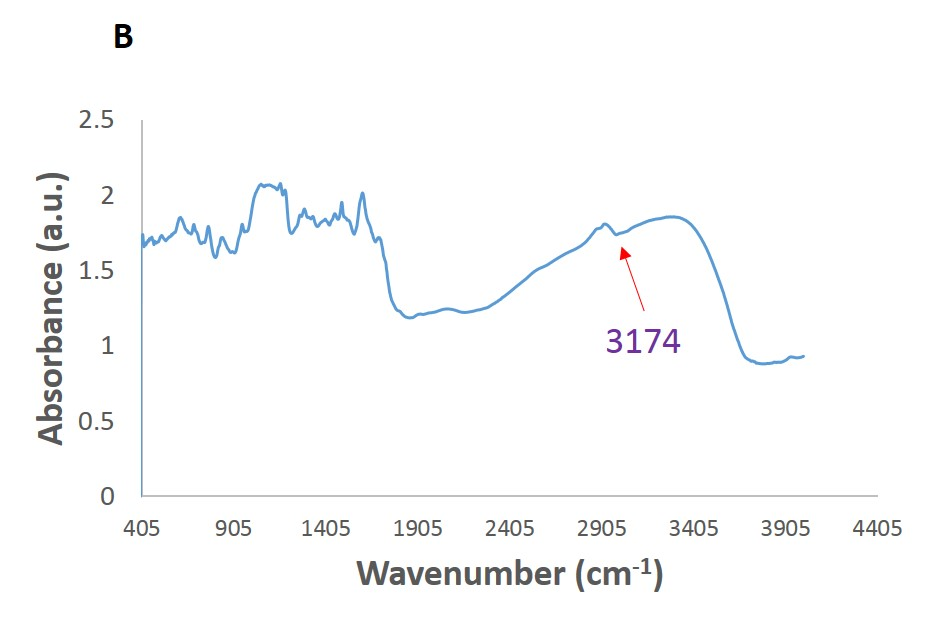
**

**
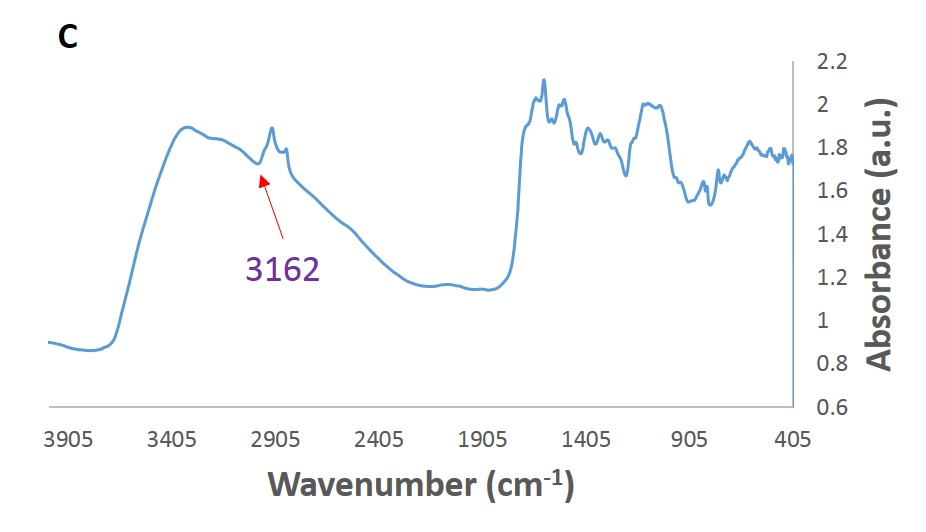

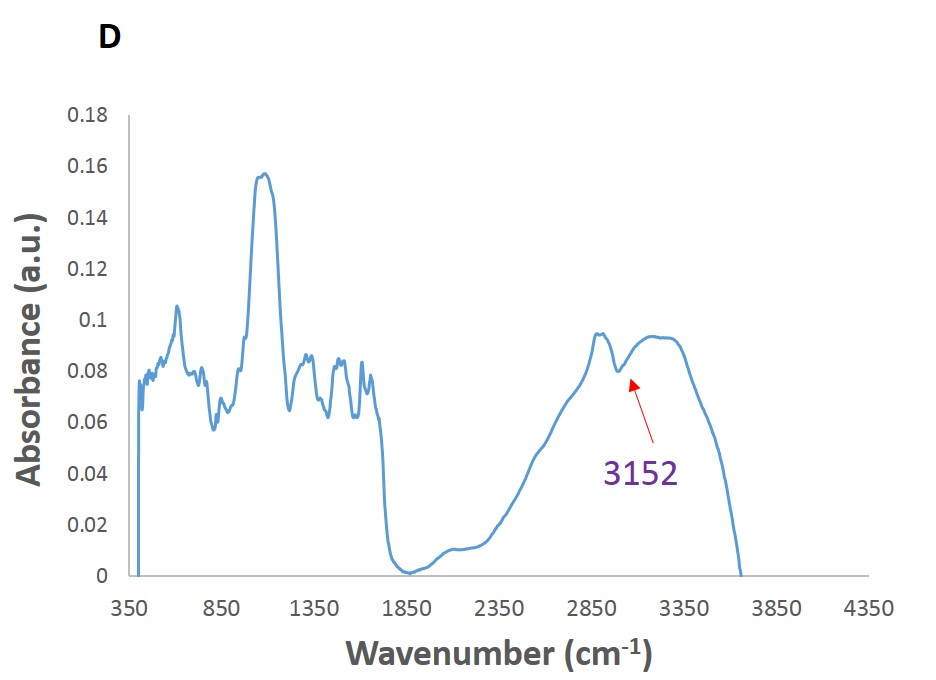
**

**Figure S4.** FT-IR spectra of folic acid conjugated polymers (A) NRFA, (B) PRFA, (C) NGFA, and (D) PGFA.

**A**

**B**

**Figure S5.** Luciferase transgene expression (RLU/mg) with plasmid pGL3.0 observed with FA*-*conjugated polymers, their corresponding parental polymers and Lipofectamine-3000 in **(A)** MDA-MB-231 (48h), **(B)** UMUC3 (48h), cells. Transgene expression was determined for polymer: pDNA weight ratios for 20:1 and 25:1. ***** = p-value < 0.05 using Student’s *t*-test; p-values were obtained by comparing RLU/mg values of each FA*-*conjugated polymer or the parental polymer with Lipofectamine-3000 under corresponding conditions. UT: untreated control. Data represent mean ± one standard deviation of three independent experiments (n=3).

**A**

**B**

**Figure S6.** Luciferase expression in MDA-MB-231 human breast cancer and UMUC3 human bladder cancer cells following delivery of pGL3.0 plasmid using different doses of Lipofectamine-3000 and P3000 reagent as per vendor’s protocol. The legend indicates volume of Lipofectamine-3000 used. (n=3).


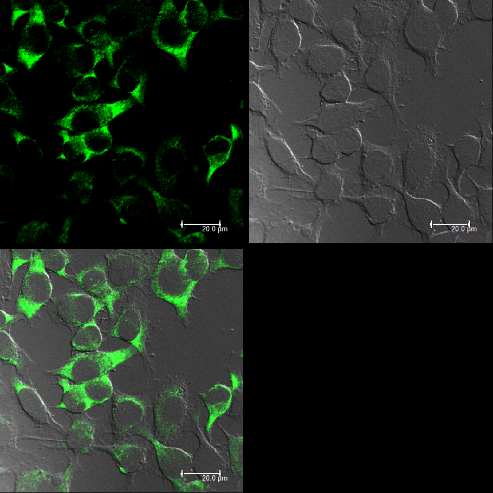


**T24 Positive control**ates folate expressionbe converted into some concentration units if available / possibile.rch Initiative (FURI) award and w


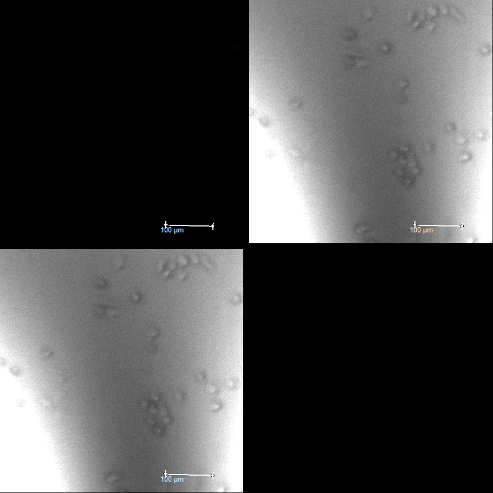


**T24 negative control**


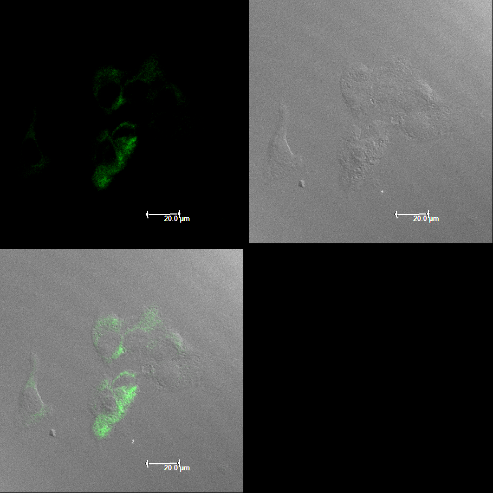


**UMUC3 cells**

**Figure S7.** Confocal images of T24 and UMUC3 human bladder cancer cells following immunostaining with folate receptor antibody alpha. Cell morphology can be visualized in phase-contrast images and the green fluorescence emission of Alexa fluor-488 indicates folate expression (top); the overlay of these two can be seen in images (bottom). T24 cells stained with primary antibody against FR-α and FITC-tagged anti-rabbit antibody (secondary) were used as a positive control and FITC-tagged anti-rabbit antibody only as the negative control.


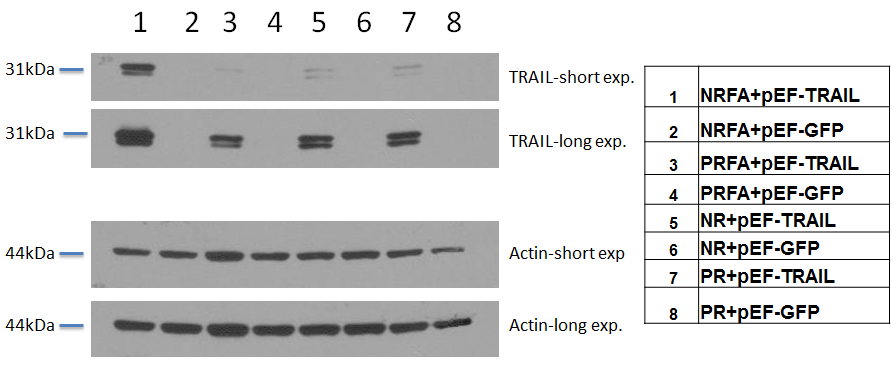


**Figure S8.** Western blot analyses showing TRAIL protein expression following polymer-mediated delivery of the pEF-TRAIL plasmid to UMUC3 cells; a plasmid expressing green fluorescent protein, pEF-GFP, was used as the control. Lane information is provided in the legend table in which the numbers in the first column indicate lane numbers in the gels and entries in the second column indicate corresponding polymer-plasmid complexes delivered.

| **Folic acid conjugated polymer** | **Calculated Folic acid/polymer mole ratio** |
| --- | --- |
| PRFA | 1.12 |
| NRFA | 0.85 |
| PGFA | 0.72 |
| NGFA | 0.83 |

**Table S1.** Extent of modification for folic acid conjugated polymers from parental polymers. The folic acid: polymer mole ratio was calculated using NMR.

| **Polymer** | **Concentration (mM) of amines by Ninhydrin assay** | | **Molar ratio**  **3.5 kDa / 10kDa** |
| --- | --- | --- | --- |
|  | **After 3.5 kDa dialysis tubing** | **After 10 kDa dialysis tubing** |  |
| PR | 3.087 | 0.32 | 9.65 |
| NR | 2.853 | 0.18 | 15.85 |

**Table S2.** Amine content analysis (Ninhydrin assay) of solution left behind followling dialysis of NR and PR polymers first through 3.5 kDa and then through 10kDa MWCO dialysis membranes indicates that most of the polymers ranged from 3.5-10 kDa in molecular weight, since almost all polymer was lost following dialysis through a 10 kDa membrane.
